# Advances in molecular biomarkers and liquid biopsy in gliomas

**DOI:** 10.1093/noajnl/vdac151

**Published:** 2022-11-11

**Authors:** Dimitrios Mathios, Jillian Phallen

**Affiliations:** Department of Neurosurgery, The Sidney Kimmel Comprehensive Cancer Center, Johns Hopkins University School of Medicine, Baltimore, Maryland, USA; Department of Oncology, The Sidney Kimmel Comprehensive Cancer Center, Johns Hopkins University School of Medicine, Baltimore, Maryland, USA

**Keywords:** circulating biomarker, circulating tumor DNA, glioblastoma, liquid biopsy

## Abstract

There have been significant strides toward understanding the molecular landscape of brain cancer. These advances have been focused on analyses of the tumor microenvironment and have recently expanded to include liquid biopsies to identify molecular biomarkers noninvasively. Moving from tissue to liquid-based analyses of molecular biomarkers has been challenging and currently, there are no approved noninvasive tests that are clinically useful. However, the emerging field of molecular liquid biopsy assay development in the neuro-oncology space has great potential to revolutionize the detection and monitoring of patients with brain cancer.

## Molecular Markers of High-Grade Gliomas

Adult and pediatric glioblastoma are resistant to treatment and invariably lethal.^1^ The current standard of care for these tumors involves surgery, radiation, chemotherapy,^1^ and/or tumor treating fields.^2^ Many promising clinical trials investigating the use of immunotherapies,^3^ laser interstitial therapy,^4^ novel biological agents, and targeted therapies such as IDH1 and BRAF inhibitors^5,6^ are underway and have the potential to improve survival in specific subpopulations. Despite the treatments available, patients with gliomas would still benefit from earlier detection of their disease and better methods for disease monitoring during treatment.

The advent of next-generation sequencing to assess alterations in DNA, RNA, and methylation has led to a strong understanding of the molecular alterations that lead to the development of brain cancer.^7^ Efforts to establish the foundation of molecular markers associated with high-grade gliomas have been possible based on analyses of a large pool of brain cancer samples that have been analyzed with all available technologies. Over the last 10 years, there have been enormous strides in understanding the molecular mechanisms of glioblastoma pathogenesis and much of the work has led to an understanding of how molecular subgroups of glioblastoma patients respond to treatment and their eventual outcome.^7^ So far the results of these efforts have identified a few key molecular alterations in gliomas such as *IDH1,2* mutations as a positive prognostic factor of overall survival, 1p19q codeletion as a hallmark of oligodendrogliomas, and *MGMT* promoter methylation as a marker of resistance to temozolomide treatment. In addition, 1p/19q codeletion status has promoted stratification of glioma patients into 2 treatment groups: Oligodendrogliomas that have a dramatic response to procarbazine/carmustine/vincristine (PCV) and radiation versus astrocytomas that are less sensitive to this regimen.^8^

**Figure 1.**
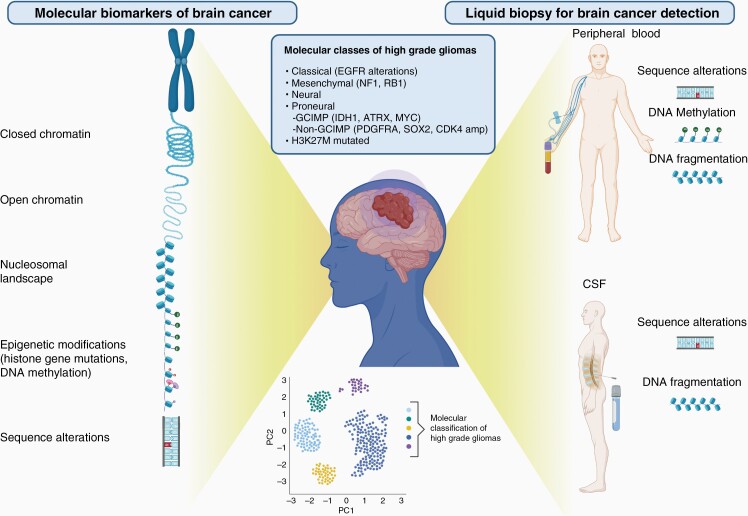
Molecular biomarkers and liquid biopsy in high-grade gliomas. Molecular analyses of the most malignant form of adult brain cancer, glioblastoma, have identified multilevel alterations in the genomic and epigenomic landscape. Clustering analysis of the RNA and methylation level data from brain cancer samples can group patients in distinct molecular subgroups with distinct clinical course and response to treatment. Liquid biopsy analyses for noninvasive brain cancer detection have leveraged the knowledge of the genomic and epigenomic landscape of brain cancer and aim to identify these cancer specific alterations in the bloodstream as well as the CSF. Figure created with BioRender.com.

Independent as well as coordinated efforts like the TCGA database have identified 6 molecular subgroups of glioblastomas based on RNA expression and DNA methylation.^7^ Each of the groups is associated with recurrent genetic alterations and each group exhibits a different genetic/epigenetic programming and treatment resistance. RNA expression analyses have identified 4 distinct glioblastoma subgroups: (1) neural, (2) proneural, (3) classical, and (4) mesenchymal. Analyses of DNA methylation cluster glioblastomas into these distinct groups and have also led to the additional subclassification of the proneural subtype based on the GCIMP (Glioma CpG Island Methylator Phenotype) and non-GCIMP phenotype (Figure 1). The proneural GCIMP phenotype has been associated with *IDH1* mutations, *ATRX* mutations, and *MYC* amplification whereas the non-GCIMP phenotype is associated with *PDGFRA*, *SOX2*, and *CDK4* amplifications. The mesenchymal subtype, in contrast, has more prevalent mutations of *NF1* and *RB1*, whereas the classical subtype has multiple alterations in the *EGFR* region (both mutations and amplifications). The role of epigenetic modifications in glioblastoma is now better understood as it has been found that chromatin dysregulation is closely associated with methylation profiles across the genome. Specifically, H3K27M midline gliomas in the pediatric population resulting from mutations to the *H3F3A* or *HISTI1H3B* genes, lead to a hypermethylator phenotype.^9^ These advances in our understanding of the molecular pathogenesis of gliomas have the potential to contribute to improved patient care, however, these markers have not yet been integrated into clinical care. One of the most promising avenues for implementing molecular markers to improve care for glioma patients is liquid biopsy research.

## Liquid Biopsies

Advances in the molecular understanding of gliomagenesis have led to recent efforts in developing assays for noninvasive detection and monitoring of brain tumors in the blood or the cerebrospinal fluid (CSF) via identification of these molecular markers. The concept of diagnosing an individual with a high-grade glioma or assessing tumor recurrence without the need for brain surgery is very appealing to patients and clinicians given the risks involved with any brain surgery. However, the development of liquid biopsy approaches for the detection of brain tumors has proven to be challenging. Recent efforts to translate the knowledge from molecular profiling of brain tumors to noninvasive approaches for the detection and monitoring of brain cancer have focused on analyses of circulating, cell-free DNA (cfDNA), circulating tumor cells (CTCs), and circulating RNAs (cRNAs). While several studies have been published on analyses of cfDNA, CTCs, and cRNAs in gliomas, none have yet progressed to clinical implementation.^10,11^ While CTC and cRNA studies have been successful in identifying additional circulating molecular markers of interest, studies of cfDNA have progressed farthest toward feasibility for clinical implementation. In this review, we highlight recent advances in liquid biopsy approaches for the detection and monitoring of high-grade gliomas based on analyses of cfDNA derived from peripheral blood (Table 1). Other reviews in this special edition focus on CSF as a source of cfDNA for liquid biopsy analyses and assessment of extracellular vesicles or circulating RNAs as biomarkers.

**Table 1.** Summary of liquid biopsy approaches assessing cfDNA for detection of high-grade gliomas

Biofluid	Molecular feature(s)	Technology	Cohort* (*n*)	Glioma subtype (*n*)	Performance		Reference
					Specificity	Sensitivity	
Plasma	*IDH1* R132 mutation	COLD and digital PCR	80	Grade II (28) Grade III (42) Grade IV (10)	100%	60%	Boisselier et al. (2010)
	*BRAF* V600E	ddPCR	9	Pleiomorphic xanthoastrocytoma (1) Grade IV (3) Metastatic tumors (5)	100%	80%	Kang et al. (2021)
	*H3F3A*, *IDH1, BRAF, ACVR1, TP53, PIK3CA mutations, MYCN* amplifications	ddPCR	27	High-grade gliomas and DIPGs (27) Pediatric HGG trial (41)	Not assessed	10%	Izquierdo et al. (2021)
	*TERT* mutations	ddPCR	157	Grade II (7) Grade III (17) Grade IV (81) Unknown (52)	90%	62.5%	Muralidharan et al. (2021)
	Sequence mutations and structural alterations	Tumor-guided targeted NGS of gene panel and PCR	23	Grade II oligodendrolgiomas (6) Grade II astrocytomas (6) Grade III (1) Grade IV (10)	100%	7%	Bettegowda et al. (2014)
	Sequence mutations	Targeted NGS of gene panel	370	Grade I (5) Grade II (25) Grade III (35) Grade IV (222) Unknown (83)	Not assessed	55%	Piccioni et al. (2019)
	cfDNA methylation	Whole genome cfMeDIP	122	IDH mutant, 1p/19q codel (28) IDH mutant, 1p/19q non-codel (42) IDH wild-type (52)	AUC>0.95		Nassiri et al. (2020)
	cfDNA methylation of *p16/(INK4a)*, *MGMT*, *p73*, and *RARβ* promoter region genes	Methylation specific PCR	10	Grade II oligodendroglioma (1) Grade III astrocytoma (2) Grade III oligoastrocytoma (1) Grade IV (6)	Not assessed	60%	Weaver et al. (2006)
	cfDNA fragmentation	Whole genome sequencing	34	Grade IV (34)	92%	59%	Mouliere et al. (2018)
Serum	cfDNA methylation	Genome-wide methylation array	149	Grade II astrocytoma (5) Grade II oligodendroglioma (5) Grade III astrocytoma (6) Grade III oligodendroglioma (5) Grade IV (27)	98%	100%	Sabedot et al. (2021)

*Cohorts reported here pertain to the number of gliomas included in the studies.

### Sources of Liquid Biopsy Material, cfDNA Biology, and Technology Development

cfDNA is shed into the bloodstream from cells as they undergo cell death and is present in all individuals. In healthy individuals, cfDNA is primarily derived from natural white blood cell turnover,^12^ while patients with cancer have a portion of cfDNA, termed circulating tumor DNA (ctDNA), which is tumor-derived. ctDNA can be identified through the detection of molecular markers such as mutations, rearrangements, copy number alterations, epigenetic modifications, and aberrant fragmentation (Figure 1).^13–18^ cfDNA can be derived from blood plasma and has a characteristic fragment size based on the preservation of fragments wrapped around nucleosomes which may be altered in patients with cancer. It is also possible to isolate cfDNA from blood serum, however larger DNA fragments from cellular genomes would also be present in the sample. CSF is also a source of cfDNA and has become another frequently assessed biofluid for liquid biopsy analyses (Figure 1).

The remarkable advancement of techniques for assessment of these molecular markers, such as PCR and next-generation sequencing (NGS) platforms has brought genomics closer to clinical application and has allowed for sensitive detection of ctDNA.^17,19^ Droplet digital PCR (ddPCR) for the detection of specific mutations is considered the gold standard for the most sensitive detection of a specific mutation yet the technology limits the number of mutations that can be assessed at once and usually requires tumor tissue sequencing for the identification of the most prevalent tumor mutation for the design of the respective ddPCR probes for tumor monitoring. NGS has become a prominent tool for liquid biopsy analyses yet the discovery of mutations in the circulation derived from clonal hematopoiesis rather than tumorigenesis has shown that mutational analyses must be confirmed by comparing to the matched tumor for mutation concordance or by subtracting mutations identified from analyses of white blood cells (WBCs).^20^

### Mutations in cfDNA

Boisselier et al.^21^ assessed for the first time the presence of *IDH1*^*R132H*^ mutation in the plasma of patients with IDH1 mutant gliomas using a PCR-based methodology. They found that plasma cfDNA from 60% of the cases examined contained the mutation unlike plasma from *IDH1WT* gliomas that did not have detectable mutant *IDH1* ctDNA. The presence of the mutation in the plasma was more frequent in higher grade gliomas and especially in the ones with higher volume of contrast enhancement on an MRI evaluation. The authors hypothesized that this association was related to increased disruption of the blood-brain barrier allowing for increased ctDNA release. Later studies that assessed the presence of *IDH1* mutations have not replicated these findings.

Specifically, the first systematic assessment of ctDNA as a biomarker for brain cancer was the 2014 Bettegowda et al. study focused on tumor-guided PCR identification of mutations in 640 patients with 1 of 14 tumor types including 27 patients with gliomas.^13^ This study highlighted key challenges of mutation detection in the cfDNA of brain cancer patients. While some tumor types such as colorectal cancer had high detection rates of ctDNA, less than 10% of patients with gliomas were detectable, the lowest detection rate across all 14 cancer types.^13^ While the study included 13 patients with an *IDH1* mutant tumor, assessment of the plasma-derived cfDNA from all 13 patients did not reveal the presence of the mutation in the bloodstream. More recently, Piccioni et al. used various commercially available targeted NGS platforms that interrogate 54–73 recurrently altered cancer genes to analyze liquid biopsies from 419 patients with primary brain tumors and detected mutations in the plasma in 55%.^22^ In this cohort genomic tumor data were not evaluated in each patient and thus plasma-derived cfDNA data were presented without confirmation of concordance with mutations in the tumor and without assessment of WBCs to rule out mutations derived from clonal hematopoiesis. *IDH1* mutation was detected in 5 patients from the cohort. Notably, 60 patients in the cohort were histologically classified as grade II or III and 222 as grade IV. This indicates the sensitivity of this technology would at best be 5/60 (~8%) of cases with an *IDH1* mutant tumor. Therefore enthusiasm for plasma mutation-based approaches has been tempered.

Another recent approach to mutation detection in cfDNA focused on the detection of *TERT* promoter mutations using a novel ddPCR assay to assess samples from patients with gliomas.^23^ Results indicated that the approach was highly sensitive as well as highly specific for the identification of tumor-derived mutations based on concordance analysis of the same mutations directly in tumor samples.^23^*TERT* mutations were detected in the preoperative plasma of 62.5% of patients with gliomas with a specificity of 90%,^23^ and longitudinal monitoring showed dynamic changes in *TERT* mutant allele frequencies that followed closely the clinical disease course including in the settings of response to therapy or tumor progression.^23^ The results of these studies highlight the challenges of cfDNA assessment, yet also indicate that detection of ctDNA as a noninvasive biomarker of brain cancer may be useful in diagnostic and monitoring settings.

Additionally, a few groups have investigated the use of ddPCR specifically for actionable mutations such as *BRAF* V600E, a mutation prevalent in craniopharyngiomas and low-grade astrocytomas. Kang et al.^24^ assessed the presence of the mutation in the tumor tissue and plasma-derived in a small cohort of 13 patients with primary and metastatic brain tumors. While the study showed that ddPCR analysis of *BRAF* V600E in the blood of patients with brain tumors is feasible (80% sensitivity [4/5 BRAF MT patients] with 100% specificity [0/4 patients had detectable BRAF mutation]), the number of primary brain tumors is quite small with the majority of the patients having widely metastatic disease. Nevertheless, the ability to detect the presence of a tumor-derived mutation in the blood is of high clinical utility as it could guide clinicians to choose the right targeted treatment for each patient at a given time point, without the need for invasive brain biopsies. A study by Izquierdo et al.^25^ attempted to develop ddPCR assays for commonly occurring mutations in pediatric gliomas. After validation of the primers for detection of *IDH1*, *BRAF*, *H3F3A*, *ACVR1*, *TP53*, *PIK3CA* mutations, and *MYCN* amplification they assessed the presence of these mutations in plasma, serum, and CSF. In their initial cohort of 32 pediatric gliomas, they were able to successfully identify glioma-related alterations in a minority of plasma samples (23%) and in the greater number of CSF samples(67%). However, upon analysis of a prospectively collected cohort of patients with pediatric gliomas (*n* = 41), no glioma-specific mutations were present in the plasma of these patients, a result which may have been in part due to low amounts of starting material (mean cfDNA extracted of 2.5 ng).

Given the challenges of ctDNA detection in the bloodstream and following the examples of other studies in lung cancer,^26^ pancreatic cancer,^27^ and colorectal cancer^28^ where other biofluids are used for liquid biopsy analysis, CSF has been assessed as a proximal source of cfDNA which may have advantages of decreased background noise and enrichment of ctDNA (Figure 1). Miller et al. evaluated CSF from 85 patients with gliomas, 46 of whom were diagnosed with glioblastoma using the MSK-IMPACT NGS panel to identify mutations.^29^ The authors found that 42 of 85 (49%) patients, including 27 of 46 (59%) patients with glioblastoma, had detectable ctDNA in the CSF.^29^ These mutations matched those present in matched primary tumor tissue indicating that this approach may be highly specific. Analyses of longitudinal CSF samples showed the gradual evolution of the genetic landscape over the time course of treatment. These results highlight the advantages of liquid biopsy analyses of CSF and the application of these methods for longitudinal monitoring of molecular markers of brain cancer.

### Methylation of cfDNA

Given the strong association of epigenetic modifications with brain tumor pathogenesis,^30^ DNA methylation of cfDNA has been investigated and has emerged as another promising biomarker accessible in circulation. One of the first research studies investigating the value of methylation in noninvasive detection of brain tumors was undertaken by Weaver et al.^31^ Although the authors assessed a small cohort of 10 patients, they demonstrated that using a methylation-specific PCR approach for interrogation of CpG sites in the promoter regions of *p16/INK4a*, *MGMT*, *p73*, and *RARβ* genes, hypermethylated CpG sites present in the tumor were also present in cfDNA in the bloodstream in 60% of the patients.^31^ Sabedot et al. used an Illumina methylation array technology for genome-wide profiling in the serum of patients with gliomas to develop a glioma-epigenetic-liquid biopsy (GeLB) score. They used a set of serum cfDNA samples from 30 patients with gliomas versus 31 non-glioma patients as a training set to find genome-wide differences in methylation specific to gliomas, designed a machine learning model to predict the diagnosis of glioma, and then applied the model to the test set of 8 gliomas and 11 non-gliomas and an additional cohort of 10 primary gliomas and 34 non-glioma samples. The results of this analysis revealed a diagnostic accuracy of 98% at a fixed GeLB threshold of 49%. They additionally looked at methylation differences in the serum of *IDH1* mutant (*n* = 19) and wild-type patients (*n* = 7). The differentially methylated areas they identified could classify *IDH1* mutant and wild-type samples at the tissue level and the serum samples from the IDH1 mutant tumor patient group clustered with the *IDH1* mutant tissue and conversely the serum samples from the *IDH1* wild-type patient group with the *IDH1* wild-type tissue. They found these methylation signatures correlated with the clinical course of the patient to mirror progression and response to treatment.^32^

NGS is also a promising tool for assessment of DNA methylation in cfDNA. The cfMeDIP–seq approach is based on immunoprecipitation of methylated cfDNA, followed by sequencing of the captured fragments.^18,33^ Application of cfMeDIP–seq in non-CNS tumor types showed that detection of early and late-stage cancers was possible as was classification of tumors based on site of origin.^18^ Nassiri et al. then applied cfMeDIP–seq to analyze patients with gliomas or with other intracranial tumors alongside a dataset of other tumor types and controls. They analyzed cfDNA from 60 patients with gliomas along with cfDNA data from 447 previously analyzed other cancers and healthy individuals, devised a machine learning model for glioma classification, and found that plasma methylation profiling could be used to distinguish gliomas from other cancer types with high accuracy (AUC 0.98) and similar performance among *IDH1* mutant and wild-type gliomas.^34^ Additionally, the authors developed a different machine learning model utilizing a cohort of 161 patients with common intra-axial and extra-axial brain tumors and showed an increased discriminatory power among the different histological subtypes examined. Interestingly, while all comparisons among different tumor types yielded almost perfect discrimination (AUC > 0.9) the performance of *IDH* wild-type gliomas had relatively poor discrimination from other cancer types (AUC 0.71) and the *IDH* mutant gliomas also had a less robust accuracy (AUC 0.82). Nevertheless, these studies are promising precursors to assessment of clinical feasibility. Subsequent research in this area will need to focus on prospective collection of samples, incorporation of matched controls, and standardization of technology platforms and blood collection protocols in order to move toward clinical implementation.

### Fragmentation of cfDNA

While interrogation of mutations and methylation in cfDNA is a natural extension of the knowledge base established by studies of brain tumor biomarkers toward applications for liquid biopsy, another emerging biomarker is cfDNA fragmentation. The principle of cfDNA fragmentation as a biomarker for cancer is based on the link between genome-wide cfDNA fragment abundance and length to the genomic and epigenomic changes occurring in the tumor. cfDNA fragmentation patterns correlate with open and closed areas of the genome as well as nucleosome distances. In healthy individuals, these structural elements of the genome are highly conserved yet are aberrant in patients with cancer.^15,16^ As cfDNA fragmentation mirrors genome-wide changes occurring during carcinogenesis, approaches which assess fragment size and representation across the genome have the potential to identify many more alterations and thereby increase sensitivity of detection compared to approaches that focus on targeted areas or one or a handful of biomarkers.

Recent analyses of cfDNA fragmentation have initially focused on assessment of cohorts outside of Neuro-Oncology and have utilized low coverage whole genome sequencing and machine learning to identify features that distinguish patients with different cancer types and individuals with cancer from those without. Mouliere et al. classified patients with cancer-based on cfDNA fragment size differences.^35^ Cristiano et al. as well as Mathios et al. assessed genome-wide cfDNA fragment features including size, representation, and copy number changes to distinguish patients with cancer from non-cancer individuals and identify the tissue of origin.^15,16^ These studies have reported AUCs above 0.9 for detection of cancer indicating that further application to brain cancer may be promising. In total 5 of 13 patients with primary brain tumors (38%) were detectable using a cfDNA fragmentation-based approach to assess cfDNA fragments and copy number alterations in CSF from patients with gliomas in a recent study.^36^ While the number of samples assessed in gliomas with this technology is small, the technology warrants further investigation to assess its potential use in Neuro-Oncology.

## Summary

In recent years cancer biology has been marked by an explosion in the understanding of the molecular pathophysiology of several cancer types including brain cancer which has led to the discovery of many cancer biomarkers. This knowledge has allowed for the development of several strategies to noninvasively identify tumor biomarkers in the circulation or in CSF (Table 1, Figure 1).

As new technologies arise, we continue to increase our understanding of tumor biology, interactions of the tumor with its microenvironment, as well as interactions of the tumor with the body at a systemic level. New molecular biomarkers are consistently being identified and moving from tissue analyses to noninvasive liquid biopsy approaches. Utilizing these biomarkers to increase our sensitivity and specificity of brain cancer detection via noninvasive approaches is at the forefront of cancer discovery. Despite the recent excitement surrounding implementation of liquid biopsy approaches for brain cancer, the field is still in its infancy. The methodology presented in this review requires additional validation steps to reveal the true potential of liquid biopsy technologies in supplementing the existing clinical algorithms in the care of patients with brain tumors. Furthermore, as molecular techniques improve and newer technologies emerge we anticipate the number of novel histology-specific distinguishing features among an array of brain tumor subtypes could allow for noninvasive molecular profiling of brain tumors. However, the implications of developing such noninvasive techniques for diagnostic and monitoring purposes are tremendous and afford the opportunity to change the way clinical Neuro-Oncology is practiced.
